# A Novel Implementation of a Social Robot for Sustainable Human Engagement in Homecare Services for Ageing Populations

**DOI:** 10.3390/s24144466

**Published:** 2024-07-10

**Authors:** Chiang Liang Kok, Chee Kit Ho, Tee Hui Teo, Kenichi Kato, Yit Yan Koh

**Affiliations:** 1 College of Engineering, Science and Environment, University of Newcastle, Callaghan, NSW 2308, Australia; 2 Engineering Cluster, Singapore Institute of Technology, Singapore 138683, Singaporekenichi.kato@singaporetech.edu.sg (K.K.); 3 Engineering Product Development, Science, Mathematics and Technology, Singapore University of Technology and Design, Singapore 487372, Singapore; tthui@sutd.edu.sg

**Keywords:** AI, elderly, embedded controller, humanoid robot, interface controller, visioning, system

## Abstract

This research addresses the rapid aging phenomenon prevalent in Asian societies, which has led to a significant increase in elderly individuals relocating to nursing homes due to health-related issues. This trend has resulted in social isolation and loneliness among the elderly, contributing to physical and mental ailments such as hypertension and cardiovascular diseases, as highlighted by the World Health Organization (WHO). To mitigate these issues, the research proposes leveraging technology, specifically the integration of robotics, to alleviate the caregiver shortage and enhance human interaction for the elderly. The novel approach involves developing a social robot designed to bridge the gap between humans and machines, combining knowledge from embedded systems, robotics, and essential soft skills for effective interaction. The authors found that this technological solution holds promise in addressing the caregiver shortage and improving the well-being of elderly individuals by reducing their sense of isolation, fostering better mental and physical health outcomes, and potentially transforming the landscape of elderly care through innovative technological applications. Future work includes expanding pilot studies and collaborating with healthcare institutions to further validate the effectiveness of the solution.

## 1. Introduction

In rapidly aging societies, particularly in Japan, demographic projections indicate a significant increase in the elderly population. In 2014, approximately 25.9% of Japan’s population was aged 65 or above, a figure anticipated to grow to a quarter of the population by 2050 [1,2,3,4,5]. Similarly, the United States has witnessed a 40% surge in individuals aged 65 or older between 2000 and 2016, with projections suggesting a quarter of the population will be in this age group by 2060 [6]. Research has underscored the profound impact of social isolation and loneliness on various physical and mental health conditions, including hypertension and heart disease [3,7,8,9,10]. In Asia, the growing prevalence of elderly individuals residing in old age homes for basic nursing [11], therapy, or rehabilitation services has led to an alarming rise in loneliness-related issues, with loneliness cited as a primary reason for suicidal tendencies among the elderly [12,13,14,15,16,17,18]. To address these critical challenges, this paper presents a novel platform that deploys a humanoid robot equipped with advanced visual recognition capabilities to discern and respond to the emotional cues of the elderly. Unlike existing solutions, our platform incorporates several innovative features that distinguish it from current technologies:

1. Advanced Emotional Recognition: Utilizing state-of-the-art machine learning and artificial intelligence techniques, particularly neural networking, our platform offers superior accuracy in recognizing and interpreting a wide range of facial expressions and emotional states in real-time.

2. Tailored Interaction Responses: The system automatically generates personalized responses aimed at uplifting the emotional well-being of the elderly. This level of customization is achieved through sophisticated algorithms that learn and adapt to individual behavioral patterns over time.

3. Integration of Soft Skills: Beyond technical proficiency, our platform integrates essential soft skills into the robot’s interaction protocols, ensuring that the interactions are not only effective but also empathetic and human-like. This holistic approach bridges the gap between humans and machines, offering a more natural and comforting presence.

4. Comprehensive Solution for Caregiver Shortage: In addition to emotional support, our platform is designed to assist with routine tasks in home care centers, thereby alleviating the escalating demand for human caregivers. This dual functionality enhances the overall efficiency and quality of care provided to the elderly.

5. Empirical Validation: Our platform has been rigorously tested and validated through pilot studies in several nursing homes, demonstrating significant improvements in both the mental and physical health metrics of the elderly participants.

Leveraging previous experience in robotic platform development and the latest advancements in AI, our research aims to provide a scalable and effective solution to mitigate the human resource shortage in elderly care and enhance the overall well-being of elderly residents [19,20]. By addressing both the technological and emotional aspects of elderly care, this project stands out as a pioneering effort in the field. I have also highlighted several key metrics below which are related to sustainability in our proposed work.

1. Advanced Emotional Recognition. The system utilizes machine learning and AI techniques, particularly neural networks, for real-time recognition and interpretation of facial expressions and emotional states. Furthermore, it enhances accuracy in recognizing a wide range of emotions, improving the robot’s interaction quality and user engagement.

2. Personalized Interaction Responses. The system generates tailored responses to support the emotional well-being of elderly users. It employs sophisticated algorithms to adapt to individual behavioral patterns, aiming to reduce loneliness and promote mental health.

3. Pilot Studies. Our future work includes conducting pilot studies and collaborating with healthcare institutions. These studies are essential for validating the robot’s effectiveness in real-world settings, ensuring it meets sustainability goals by being practical and beneficial in actual use cases

4. Miniaturization and Efficiency. We have made some good progress to miniaturize the robot’s integrated structure to fit within a compact frame, making it more portable and efficient. This proposed design approach aligns with sustainability by potentially reducing material use and energy consumption.

The above metrics are aimed at creating a sustainable and effective solution for elderly care through advanced technology, user-centered design, and ethical practices. This section highlights the specific contributions and innovative aspects of the platform, providing clear justification of its novelty and superiority over existing solutions. The structure of this paper is as follows: Section 2 will introduce the extensive literature review conducted; Section 3 will discuss the methodology employed; Section 4 will present the design and development of the proposed robot; Section 5 will conclude this research work; and Section 6 will present the feasible future for this proposed work.

## 2. Literature Review & Context

To ensure comprehensive coverage when devising a robotic solution with substantial human interaction, extensive research was conducted in this domain. Initially, emphasis was placed on fostering effective companionship between humans and robots. This entails cultivating a deeper level of interaction imbued with meaning and, importantly, attractiveness without sacrificing reliability. For instance, Puffy, a robot highlighted in previous studies [8], adeptly identifies user gestures, facial expressions, movements, and emotions, translating these inputs into intentions and delivering engaging experiences through multisensory systems [9,10,11,12,13]. Research has also revealed a preference among the elderly for relatively small-sized humanoid social robots [14,15,16,17,18,19], suggesting that such dimensions evoke sentiments akin to the closeness felt with a friend or child. Advancements in AI models, particularly through neural networks like CNN (convolutional neural network) have empowered companion robots like NICO to perceive and interpret various user expressions, associating them with perceived emotions and adjusting their own facial expressions accordingly [9]. Effective interaction between humans and mechanical robots hinges on the accuracy of capturing and classifying emotions from facial expressions, as demonstrated by recent studies [20]. Additionally, incorporating body language cues into social robots enhances emotional interpretation and fosters natural interaction [16]. Biologically inspired robotic models, such as the CONBE robot proposed by [16], leverage topological consciousness and adaptive resonance theory to generate emotions based on memory and external stimuli, resulting in natural interactions imbued with social empathy and sympathy functions. Analogous to the relationship between individuals and their animal companions, such as dogs, social robots designed for companionship deserve equal respect and consideration. Nonetheless, challenges persist in achieving lifelike attributes, emotional expression, and social personality in robots [21], exacerbated by media portrayals that often cast robots in a negative light, perpetuating apprehensions and reinforcing the importance of avoiding the “Uncanny Valley” in humanoid robot design [2]. Ethically navigating the evolving dynamics of human–robot relationships can significantly enhance the well-being of the elderly by fulfilling their emotional needs and fostering greater happiness. Recognizing the importance of mental well-being in older age, innovative solutions like ElliQ [22,23] have emerged in the industry. However, these solutions, though touted for their verbal and proactive interaction, often rely on touchless interfaces akin to encapsulated tablets or PCs, necessitating direct responses from the elderly. Other solutions such as SAM [24,25], Zora [26,27], and iPal [28,29] exist, yet they overlook the emotional aspect crucial for elderly care. In the context of the research paper, touchless interfaces necessitate direct responses due to the design and interaction mechanisms they employ. These interfaces, often resembling encapsulated tablets or PCs, rely on verbal and proactive engagement, where the elderly user is required to respond directly to prompts or interactions initiated by the device. This interaction method requires the elderly to be actively engaged and respond vocally or through specific gestures, which can sometimes be challenging due to cognitive or physical limitations. Thus, while touchless interfaces aim to simplify interaction, they inherently demand a level of direct response that can be difficult for some elderly users to maintain consistently. This necessitates designing more adaptive and sensitive interaction methods that can cater to the varied capabilities of elderly individuals.

## 3. Methodology

This project delves into addressing the emotional well-being of elderly individuals by analyzing their facial expressions. The formulation of problems and potential solutions guiding the project’s development encompasses several key considerations. One pivotal area of focus integrates recent advancements in facial recognition within the realm of AI, alongside embedded systems for the robotics platform. Essential to the design of any robotic platform, especially those interacting closely with humans, are key factors such as user interface and feedback mechanisms. In the context of this project, the “users” are elderly individuals residing in home care centers. Given the diverse challenges encompassing health issues and potential language barriers among the elderly, designing a universally intuitive interface is paramount, minimizing the learning curve. Thus, the chosen user interface for this project revolves around capturing emotions displayed on the user’s face, as research suggests exploring emotional states in the elderly effectively addresses their needs [18]. Emotions are classified into four distinct categories, and trained models are employed for this purpose. Additionally, the feedback system is a crucial aspect. Traditionally, in the realm of home care centers, caregivers provide immediate feedback to tend to the needs of the elderly. However, this project shifts the focus to the emotional realm of the elderly. Rather than relying on the time and effort of human caregivers, a humanoid robot is employed to fulfil this need, offering specially curated content through three distinct feedback media: audible voice, expressive eye movements, and human-like movements within a cute, baby-sized humanoid robot. The methodology for facial expression analysis in this proposed work involves using machine learning models for classification and inference of emotions. The AI unit, utilizing the Nvidia Jetson Nano platform, was chosen for its cost-effectiveness and sufficient power to handle the required tasks. The camera linked to the Nvidia Jetson Nano conducts facial scanning for emotion recognition. The model was trained using the open-source face and emotion code developed by Priya Dwivedi. The trained model categorizes emotions into four classifications. For training and validating the models, our proposed work employed standard machine learning techniques such as data collection, pre-processing, model training, and validation. This approach ensures that the model is capable of accurately recognizing and classifying the emotions displayed by elderly users, facilitating appropriate and empathetic responses from the robot.

## 4. Design & Development

This proposed work comprises of three major sub-systems which include humanoid robot, interface controller, and embedded AI and visioning system.

### 4.1. Humanoid Robot

This segment represents the most intricate phase of the entire project, encompassing various sub-systems that demand meticulous attention. Prior to devising the robotic platform, establishing clear objectives for the final product was imperative. Initially, the research focus was on lower primary to kindergarten age groups. This decision was influenced by collaborative efforts with the team, who contributed to media creation, including children’s stories, art drawings, and games for the website. The robot behaviors and stories were simple and fun bedtime stories we found on the website and in the children’s YouTube channel.

Once the target audience was firmly established, key features of the robotic platform were being outlined, prioritizing human-like movement within a child-sized framework, audible voice and sound capabilities, and expressive eye expressions. The adaptation of a robot initially designed for children could be aimed at the elderly; the rationale is provided below.

1. Similar Needs. Both children and the elderly often require companionship, assistance, and entertainment. Robots designed for children might already possess features that can be beneficial to the elderly, such as simple communication interfaces, reminders, or interactive capabilities.

2. Ease of Adaptation. Many aspects of child-oriented robots, such as friendly appearances, gentle voices, and simplified interfaces, can easily translate to designs suitable for the elderly. These characteristics can make the robots approachable and user-friendly for older adults.

3. Effective Cost Management. Developing a new robot from scratch can be time-consuming and expensive. Adapting an existing robot designed for children can be a more cost-effective solution, especially if the core functionalities align with the needs of the elderly population.

4. Ease of Customization. While the base technology may be designed for children, it can be customized and enhanced to better suit the needs of the elderly. This customization can include features like medication reminders, emergency assistance, or cognitive stimulation exercises tailored to older adults.

**Figure 1:**
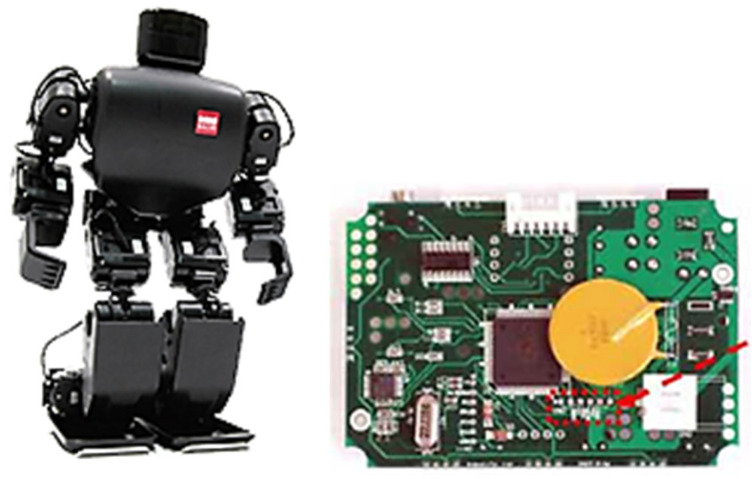
Original humanoid robotic platform and its 8-bit embedded controller.

Subsequently, attention should be focused on sourcing a robotic platform that would resonate well with this age group. RoboBuilder’s platform, a 30 cm tall humanoid robot, depicted in Figure 1, emerged as a potential candidate. However, while it boasted 16 Degrees of Freedom (DoF), it fell short in terms of its embedded controller—a mere 8-bit Atmel microcontroller—capable only of executing pre-processed motions curated from a computer.

**Figure 2:**
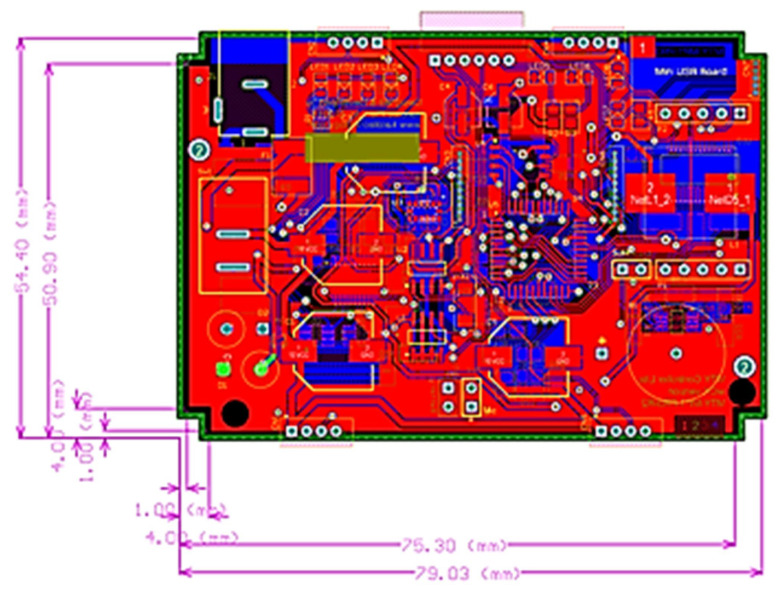
Final designed robotic controller for the humanoid robot called Matanya.

The board layout depicted in Figure 2 represents the third iteration, with the initial two iterations serving as prototypes. This iteration shares the same form-factor as the original controller to seamlessly integrate it into the controller’s enclosure and body frame. This design incorporates features capable of fulfilling all three primary objectives outlined earlier. The chosen main controller is a low-powered eight-core RISC processor, specifically the P8X32A by Parallax. Given that content delivery involves three distinct media—audible sound/voice, eye expression, and robotic movement—this multi-core microcontroller is ideally suited for executing multiple processes simultaneously.

**Figure 3:**
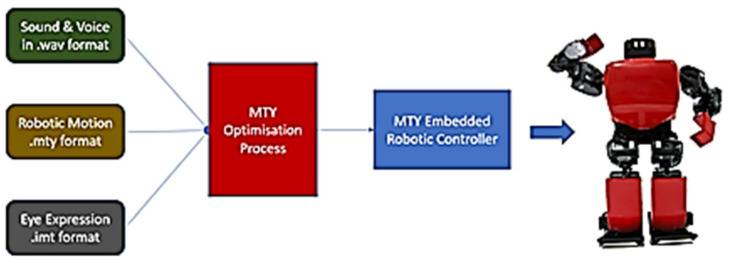
The medium process flow.

**Figure 4:**
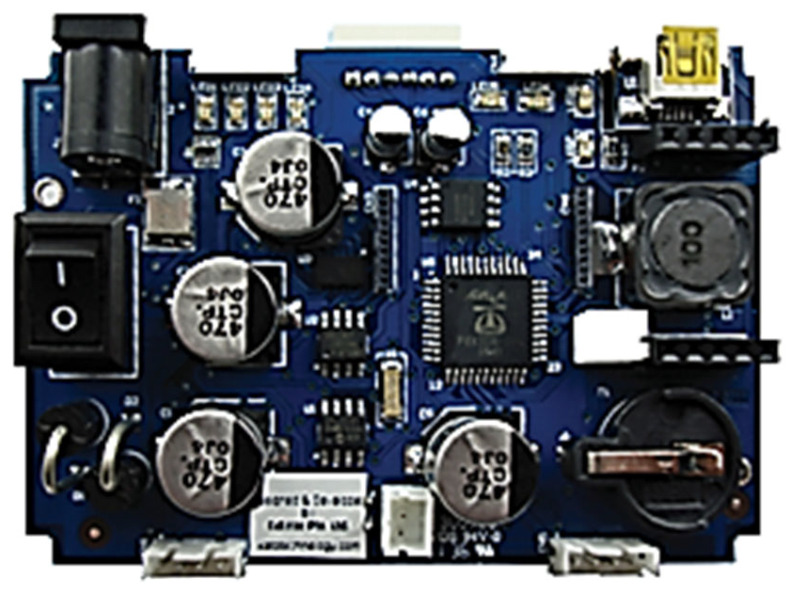
Completed fabricated robotic controller.

The data feeding process into the controller necessitates pre-processing software, which was personally developed using Java. Ensuring synchronized execution of the media on the robotic controller is crucial. The process flow block diagram, illustrated in Figure 3, delineates this workflow. Additionally, the final iteration of the controller, depicted in Figure 4, was meticulously hand-soldered.

In addition to the major features required, several other onboard features for better monitoring control and future scaling of additional features have been added.

- MMA7455, 3-Axis Accelerometer IC
- LTC4011CFE, NiMH Battery Charger IC
- DS1307Z, Real-Time Clock IC
- FT232RL, FTDI Full-speed USB Controller IC
- LM4990MM, Mono Analog Class-AB Audio Amp IC
- Microphone circuit and connector
- Speaker circuit and connector
- Over-voltage and reverse polarity protection
- MicroSD circuit and socket
- Multiple SMD LED as indicators for charging, USB, power and debug
- 4 × ports connectivity to the 16 smart servos
- 1 × port connectivity to the 0.96″ OLED eye expression display unit
- Daughterboard for 2nd microcontroller as extension socket

**Figure 5:**
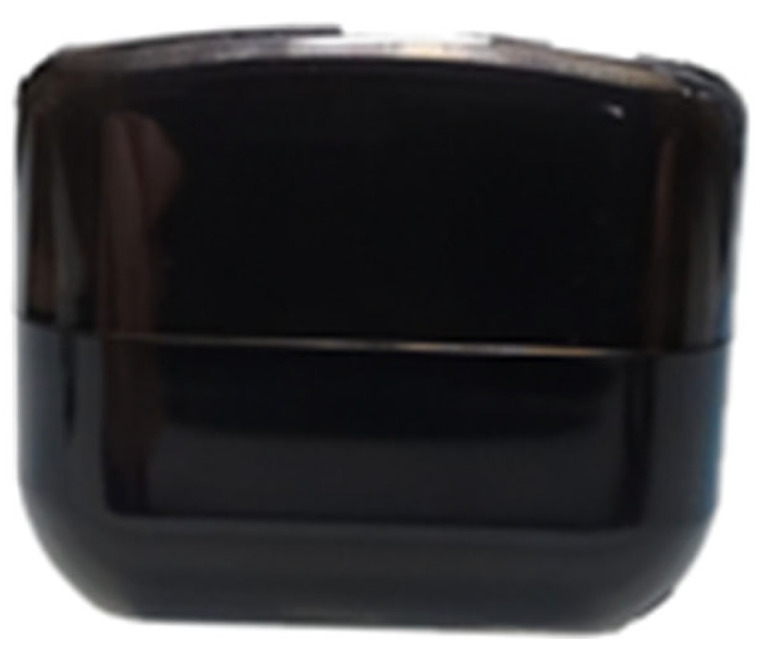
Original empty head unit.

The overall head unit from the original robotic platform is merely a hollow shell, as shown in Figure 5 below.

**Figure 6:**
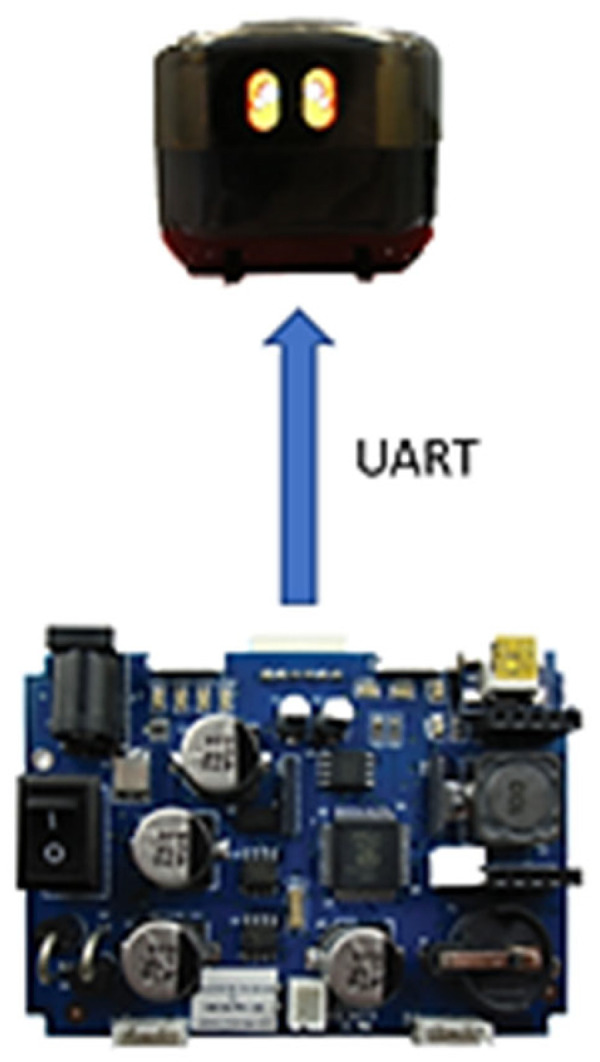
Embedded 0.96″ OLED screen inside the head unit.

One of the primary goals was to incorporate expressive eye movements into the design. To achieve this, a small screen was necessary. The 0.96″ OLED unit from 4D, chosen for its onboard microcontroller featuring a built-in graphics chipset—the GOLDELOX-GFX2 processor. This processor utilizes its proprietary programming language, known as 4DGL (4D Graphics Language). After familiarizing with this language, the necessary code and libraries for generating eye expressions will be written, generated, and stored in the unit’s local storage. Consequently, the data packets transmitted from the main robotic controller to the microcontroller for controlling the eye expressions are extremely compact, requiring just 4 bytes per expression. The process block diagram illustrating this workflow is provided in Figure 6.

**Figure 7:**
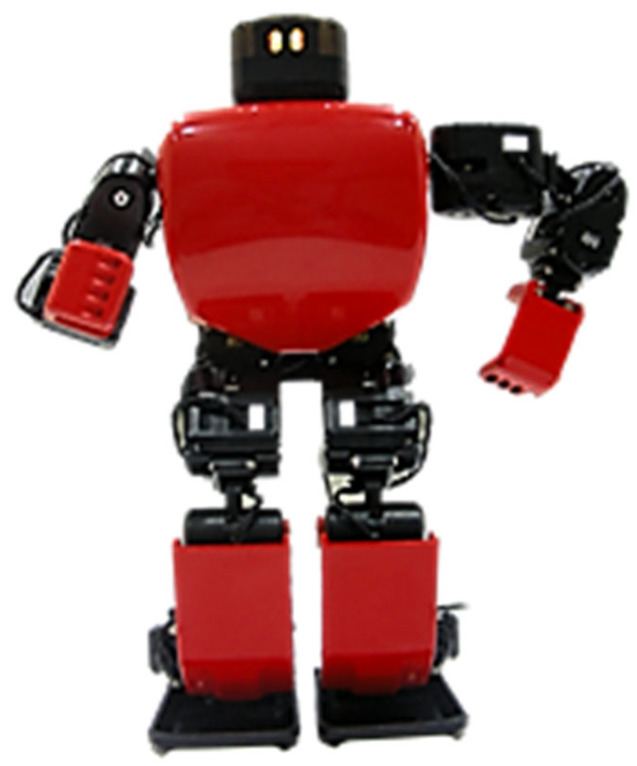
One of the units that was showcased in the exhibition.

The communication between the head unit and the robotic controller occurs via UART at a speed of 115,200 bps. The eye expressions are crafted meticulously from scratch. This process resembles sketching, akin to drafting a static comic strip. Each pixel within these expressions contains 8-bit color information, meticulously stored within the NAND flash storage unit housed in the head unit. Following the completion of the design and fabrication of these subsystems, the integration process unfolded seamlessly, as depicted in Figure 7.

**Figure 8:**
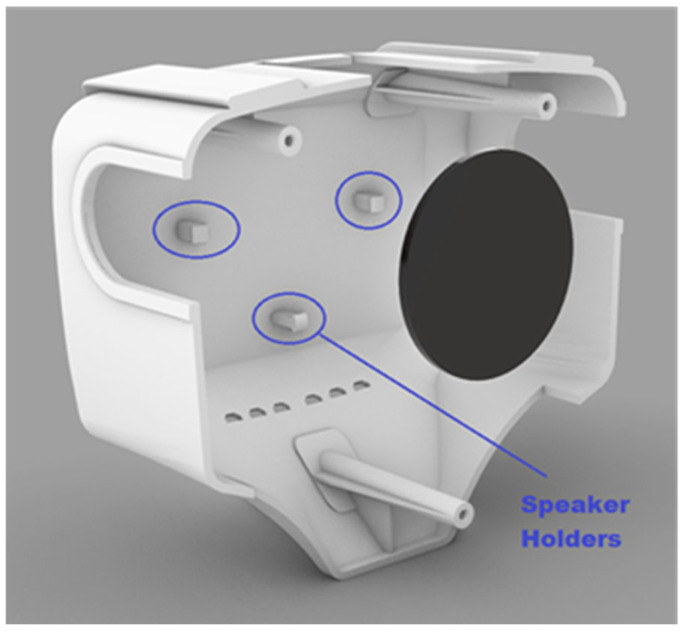
Inside of the new body with speaker holders.

The platform made its debut at the International Robotic Exhibition in Tokyo, Japan, back in 2011. However, for the purposes of this project, a redesigned body enclosure will be required. Primarily focusing on functionality, three holders have been incorporated to accommodate a sleeker 5 W speaker, as illustrated in Figure 8.

**Figure 9:**
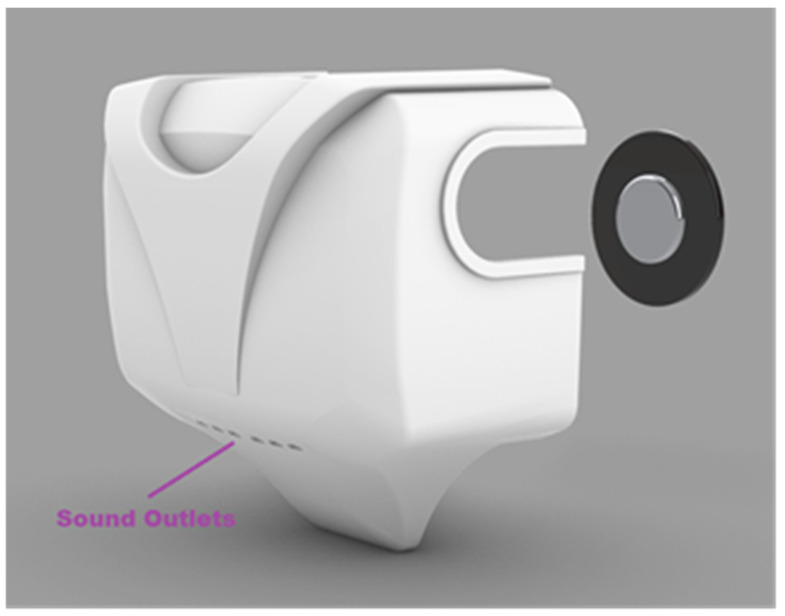
New body shell.

Additionally, small outlets in the redesigned body have been designed to facilitate the exit of sound waves—an element absent in the previous design. This adjustment not only enhances functionality but also contributes to an improved aesthetic appearance. Utilizing Rhino3D, the new body design is crafted, showcased in Figure 9, featuring six 6 × 4 mm outlets angled at 45° downwards. This strategic positioning ensures that the sound waves exit from the front rather than the back, thereby providing clearer feedback to the user.

**Figure 10:**
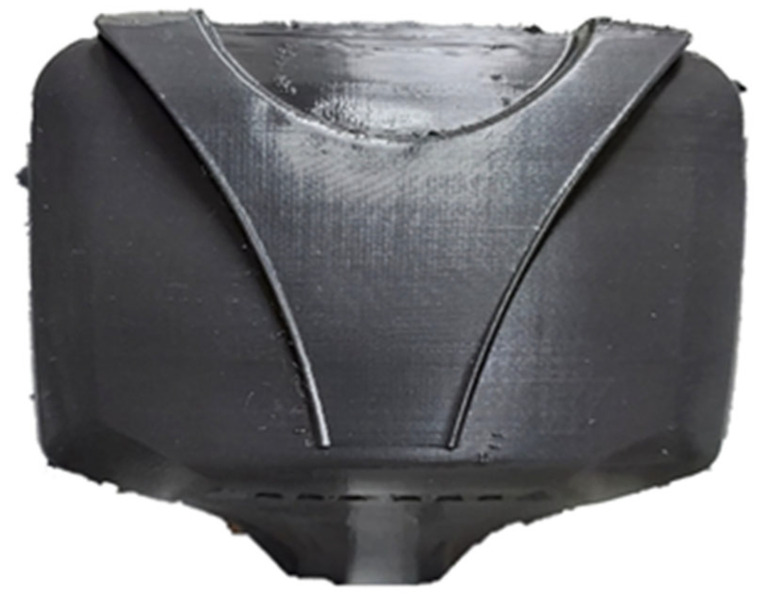
3D printed version of the newly designed body enclosure.

Figure 10 shows the 3D printed version of the design.

### 4.2. Interface Controller

**Figure 11:**
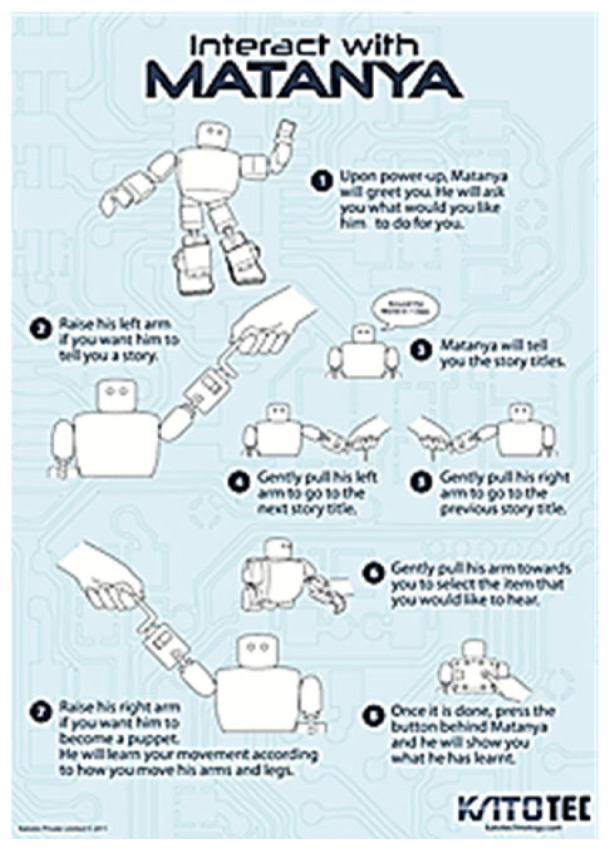
Poster to show visitors at the iREX 2011 on how to interact with MTY.

The completed robotic platform takes input from users through the selection of the robot’s hands as shown in Figure 11.

**Figure 12:**
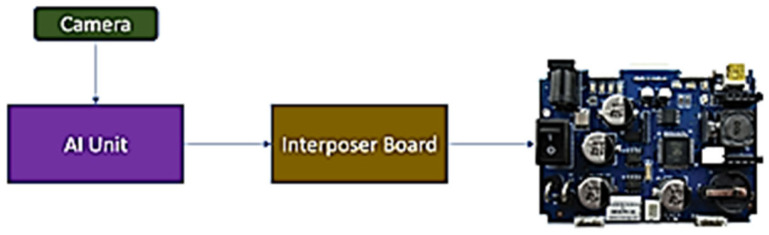
Interposer board interfacing with the AI unit and MTY robotic controller.

Consequently, the code on the robotic platform has to be modified to receive instructions from the onboard Mini-USB socket instead of relying on the robot’s manual selection. As the robotic controller operates solely on a single P8X32A multi-core processor, there was no additional code space available for expansion. Thus, an interposer board is necessary to manage the feedback from the AI unit before transmitting commands to the robotic controller. The process flow block diagram illustrating this workflow is depicted in Figure 12.

Observations were performed on the interaction between the robot and the elderly users and shown in Table 1 below. Facial responses were verified with verbal questions to the elderly users. The initial response from the robot provided a secondary response from the elderly users that would validate the initial response. It was a simple trial with our family members displaying a sad and angry face expression.

**Figure 13:**
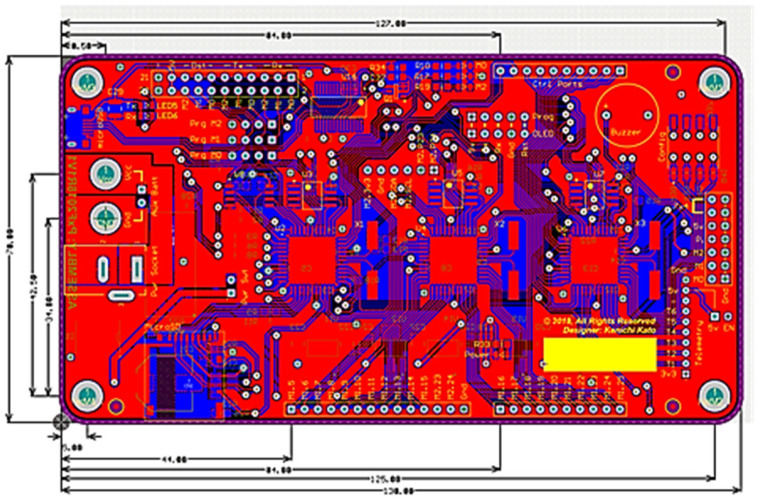
3xP8X32A multi-core embedded system.

**Figure 14:**
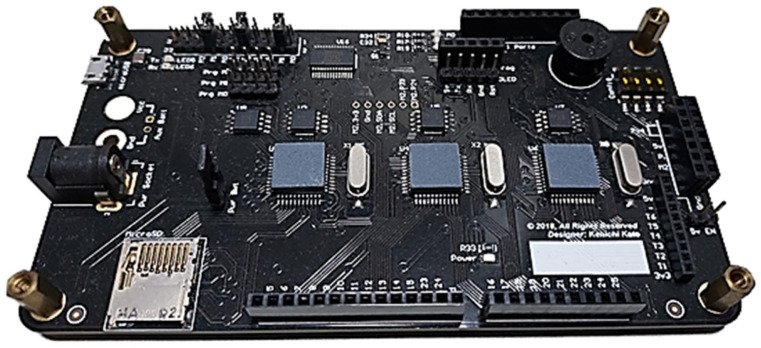
Fully mounted 3xP8X32A board that was used in this project.

Given that the functions required in the interposer board are not particularly resource-intensive, to economize costs for this project, an embedded system designed in 2018 is being deployed as depicted in Figure 13. The fully assembled unit is showcased in Figure 14. This board was initially conceived to facilitate co-development in a multi-user environment, with each group focusing on distinct tasks while adhering to a common protocol, allowing separate processors to communicate with each other seamlessly. Ultimately, the integration of these isolated yet interdependent codes will resolve a complex problem. For this proposed work, only one processor will be utilized.

### 4.3. Embedded AI and Visioning System

**Figure 15:**
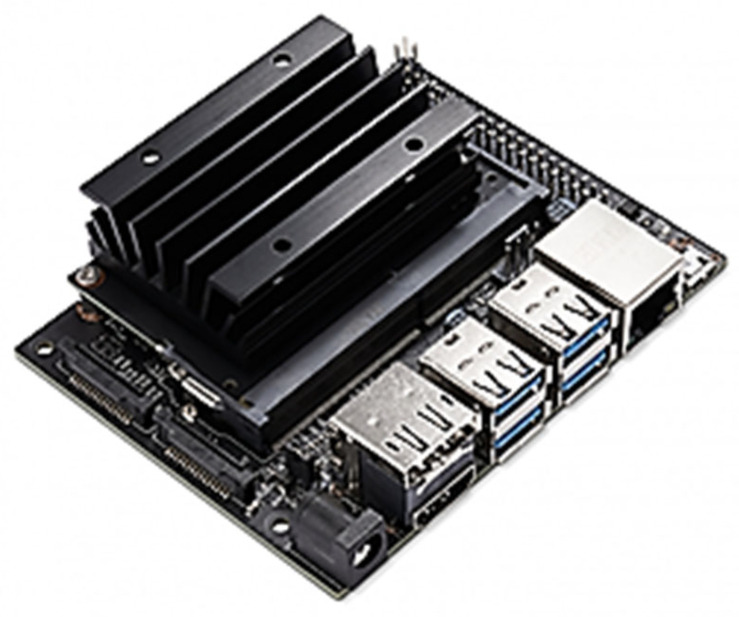
Selected AI platform for this project, Nvidia Jetson Nano Python.

The final significant sub-system involves the AI unit paired with a camera to conduct facial scanning for emotion recognition. Such tasks necessitate machine learning to train the model for classification through inference. For the AI hardware, Nvidia Jetson Nano is used as depicted in Figure 15. This choice is both cost-effective and sufficiently powerful to handle the tasks required for this project, boasting the following specifications:

- CPU: ARM Cortex-A57 × 4
- GPU: 128-core Maxwell GPU
- RAM: 4GB LPDDR4

Developing and implementing a vision system for a robot is a multifaceted process that involves several crucial steps and considerations. In the context of the proposed work, the vision system is tasked with a range of functionalities, including object, people, and emotion detection. To begin, we leverage common software frameworks tailored for vision systems, such as OpenCV, TensorFlow, and PyTorch. These frameworks provide robust tools and algorithms essential for processing visual data efficiently. Next, we curate a diverse dataset of images or videos that align with the objectives of the vision system. This dataset encompasses various real-world scenarios and conditions, ensuring the system’s adaptability and robustness. For object detection, we might use datasets like COCO (Common Objects in Context) and ImageNet. For people detection, datasets like PASCAL VOC and WIDER FACE are valuable, while for emotion detection, the FER-2013 (Facial Expression Recognition) dataset or AffectNet are often utilized. Each data point is meticulously annotated with labels or annotations corresponding to the objects, features, or emotions of interest, laying the groundwork for effective model training.

Before training begins, it is crucial to preprocess the collected data to enhance its quality and suitability for training. This involves a series of tasks, including resizing images, noise reduction, lighting correction, and data augmentation techniques. By augmenting the dataset with synthetic data, we bolster its diversity and improve the model’s ability to generalize across different scenarios. With the pre-processed data in hand, we proceed to train the selected models using state-of-the-art algorithms, such as Convolutional Neural Networks (CNNs) and their advanced variants like YOLO (You Only Look Once) for object detection, Faster R-CNN for people detection, and ResNet or VGG models fine-tuned for emotion recognition. Adjusting hyperparameters and optimization strategies, such as learning rate and batch size, is essential to optimize performance. Through iterative training cycles, we strive to achieve satisfactory accuracy and robustness in the model’s predictions. Validation of the trained models is conducted using separate datasets to evaluate their performance comprehensively. Metrics such as precision, recall, F1 score, and confusion matrices provide quantitative insights into the system’s accuracy and generalization ability. Precision and recall assess the accuracy of positive predictions, while the F1 score balances these metrics to give a single measure of performance. Confusion matrices offer a detailed breakdown of correct and incorrect classifications across different classes.

**Figure 16:**
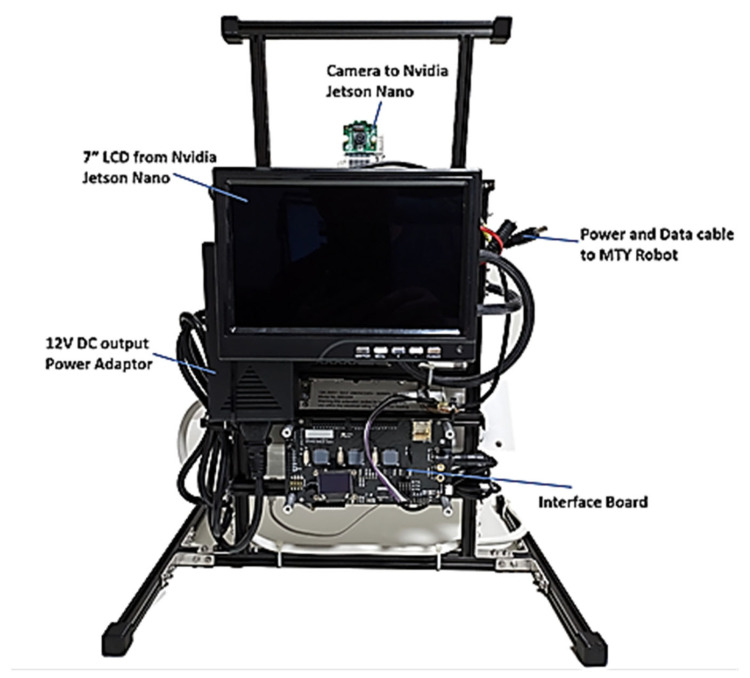
Front of the Integrated Structure.

**Figure 17:**
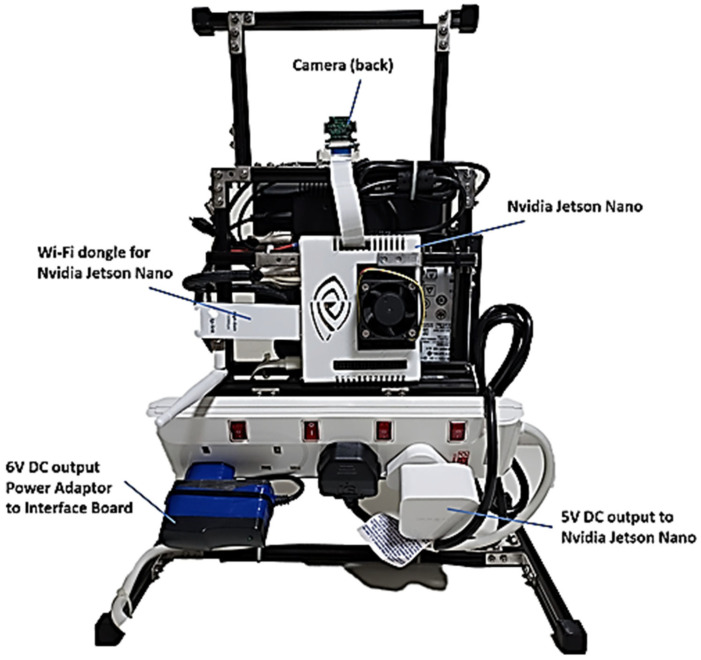
Back of the Integrated Structure.

**Figure 18:**
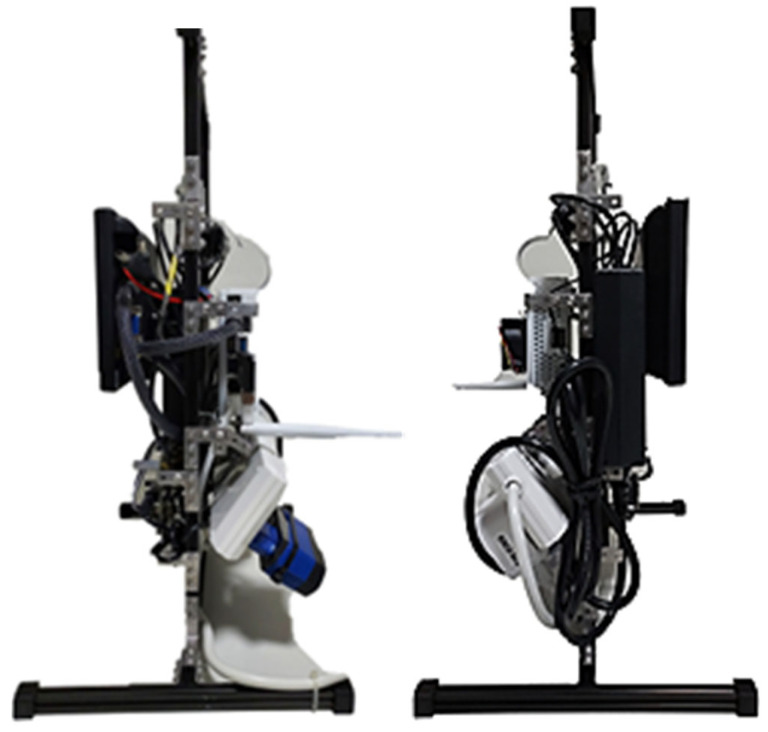
Sides of the Integrated Structure.

Integration of the developed vision system with the robot’s hardware and software platform is the final step in the process. This integration ensures seamless compatibility and communication between the vision system and other components of the robot, including actuators, sensors, and control systems. By aligning these components cohesively, we empower the robot to perceive and interact with its environment intelligently and autonomously. To securely keep the unit in place, a structure to house all these elements is implemented as shown from Figure 16, Figure 17 and Figure 18.

**Figure 19:**
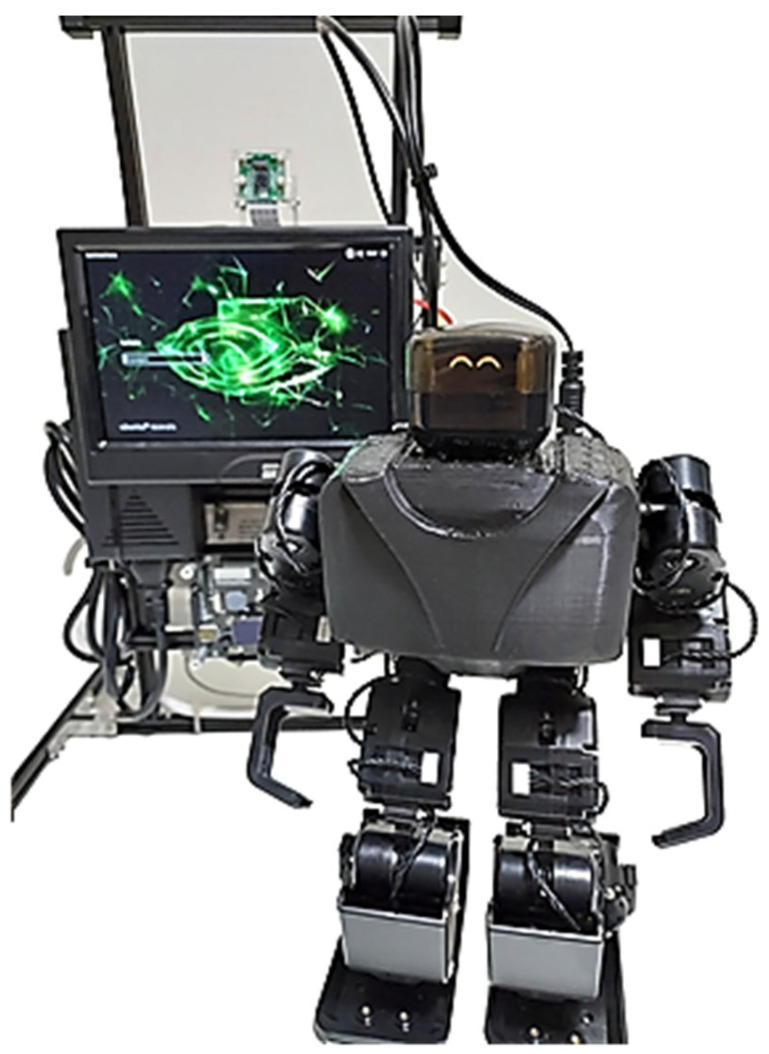
Integrated Solution with the MTY robot.

The MTY robot will be placed in front to provide feedback to the elderly as shown in Figure 19.

**Figure 20:**
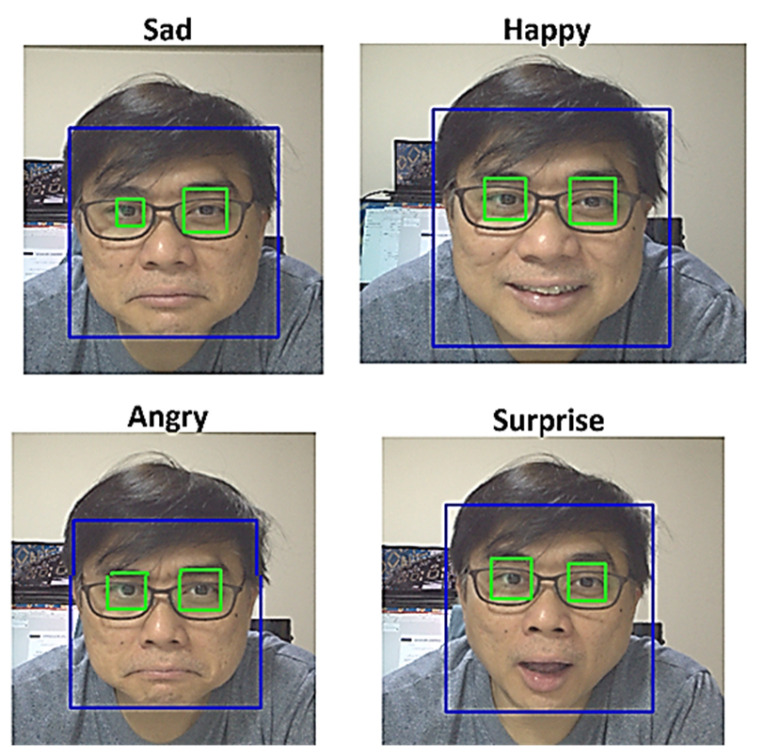
4 types of expressions the trained model will recognized.

The camera is linked to the Nvidia Jetson Nano through the CSI socket. Positioned above the MTY robot, the camera creates the illusion of the MTY robot “observing” the user. On the Nvidia Jetson Nano, the open-source face and emotion code developed by Priya Dwivedi is being used to train the models. Four classifications were trained for inference. An example of an image captured by the Nvidia Jetson Nano is depicted in Figure 20. Our proposed work classifies emotions into four categories: happy, neutral, sad, and angry. These specific emotions were chosen because they represent a broad spectrum of basic human emotional states. This categorization helps in effectively recognizing and responding to the user’s emotional needs, which is crucial for the robot’s interaction with the elderly. The selection of these emotions is likely due to their fundamental nature in human emotional expression, making them easily recognizable and significant for meaningful social interactions. This allows the robot to engage with users in a manner that is empathetic and responsive to their emotional states, thereby enhancing the overall user experience and promoting emotional well-being.

## 5. Discussion

To balance the trade-offs between miniaturization and the functionality of the robot involves several strategies. The key considerations and planned approaches include:

1. Redesigning Components for Compactness. Each component within the Integrated Structure, such as the Nvidia Jetson Nano platform, the camera, and the interface controller, will be redesigned to minimize size without compromising performance.

2. Advanced Integration Techniques. Using techniques like system-on-chip (SoC) designs, which integrate multiple functionalities into a single chip, can significantly reduce space requirements while maintaining or even enhancing performance.

3. Efficient Housing Design. Developing a custom housing that precisely fits all components while maximizing internal space efficiency. This involves advanced design and 3D printing technologies to create compact yet functional prototypes.

4. Power Management. Compact Power Solutions by exploring compact and efficient power solutions such as smaller, high-density batteries and power-saving technologies to ensure the robot remains operational for extended periods without needing large power supplies.

5. Scalable Design. Designing the robot to be scalable, allowing for additional features to be added as technology advances or as needed, without compromising the current compact design.

6. Performance Testing and Iteration. Incorporating user feedback, especially from the elderly, to understand which functionalities are most valued and where compromises can be made without significantly affecting the user experience.

By employing these strategies, the project aims to create a robot that is both compact and highly functional, ensuring it meets the needs of elderly users while maintaining high performance and usability. To also ensure that the robot respects the dignity and autonomy of elderly users, the proposed work outlines several strategies.

1. User-Centered Design. The design and functionality of the robot are tailored to meet the specific needs and preferences of elderly users. By involving elderly users in the design process and gathering their feedback, the project aims to create a robot that genuinely respects and addresses their needs.

2. Promoting Independence. The robot is designed to assist rather than take over tasks, encouraging elderly users to maintain their independence. It provides support in areas where assistance is needed while enabling users to perform tasks on their own whenever possible.

3. Personalization and Customization. The robot offers personalized interactions and responses based on the preferences and habits of each user. This personalization helps the robot to be more respectful and considerate of the user’s individual lifestyle and choices.

4. Ethical Programming. The robot is programmed to follow ethical guidelines that prioritize the dignity and autonomy of users. This includes ensuring that the robot’s behavior is always respectful and that it aids in a manner that enhances the user’s sense of self-worth and control over their own life.

These strategies are designed to create a supportive and respectful environment for elderly users, ensuring that the robot enhances their quality of life. For the AI expression recognition method, we employed the standard based model, face recognition from Adam Geitgey (MIT License) in ubuntu distribution’s repository, placing a face detection, face recognition, and emotion detection process as reference from https://github.com/priya-dwivedi/face_and_emotion_detection, accessed on 14 August 2023. The emotion detection model needs to be trained to be effective. However, there are specific technical challenges faced in the miniaturization of our proposed work. Space constraints present a significant challenge as the current Integrated Structure is too large to fit within the humanoid robot’s frame, especially given the need to house the Nvidia Jetson Nano platform, camera, interface controller, and power supply within a limited space. Ensuring that all necessary components fit and function correctly in a smaller space requires careful planning and design, necessitating the miniaturization of each component, including the AI hardware, visioning system, and interface controller, without compromising performance. Additionally, reducing the size of the Integrated Structure involves finding a compact yet efficient power supply solution that can sustain the robot’s operations. To address these challenges, the proposed solutions will be presented here. Redesigning components for compactness involves revisiting each part with a focus on reducing size, which might include using more compact versions of existing components or redesigning parts for more efficient spatial fitting. Concurrently, developing a new housing design that can accommodate all necessary components in a more compact form factor is essential, leveraging advanced design software and 3D printing technologies to create a prototype that meets size requirements. Additionally, optimizing the internal layout and wiring of components will minimize space usage and improve efficiency, requiring careful planning of component placement and wire routing to avoid clutter and maximize available space. Our proposed work emphasizes minimizing the learning curve through a straightforward and intuitive user interface, utilizing clear emotional cues and simple robot responses. It employs multisensory interaction with visual, auditory, and motion-based feedback, accommodating various sensory preferences and abilities to ensure inclusivity and accessibility. The robot’s emotion recognition system captures and analyses user emotions, reducing the need for complex manual interactions. Simplified feedback mechanisms, including audible voice, expressive eye movements, and human-like movements, make interactions easily understandable and engaging for elderly users. Future work involves pilot studies and collaborations with healthcare institutions to validate the robot’s effectiveness in real-world settings. Additionally, expanding the range of emotional expressions the robot can recognize ensures it meets the emotional needs of the elderly through iterative testing and technological upgrades. These efforts collectively aim to sustain the quality of educational learning and ensure practical, beneficial outcomes in elderly care.

## 6. Conclusions

To ensure the user interface is universally intuitive for the elderly, considering potential variations in cognitive and physical abilities, the following strategies have been implemented in the proposed work:

1. Emotion Recognition for Interaction. The user interface will revolve around capturing and analyzing the emotions displayed on the user’s face. This method reduces the need for complex manual interactions, making it more accessible for users with varying cognitive and physical abilities.

2. Simplified Feedback Mechanisms. The robot will employ three primary feedback media: audible voice, expressive eye movements, and human-like movements. These modes of communication are designed to be easily understandable and engaging for the elderly, ensuring that they do not have to navigate complex interfaces or touchscreens.

3. Minimizing the Learning Curve. The design focuses on minimizing the learning curve by creating a user interface that is straightforward and intuitive. This includes using clear, easily recognizable emotional cues and straightforward responses from the robot to maintain simplicity and effectiveness in interactions

4. Multisensory Interaction. By utilizing multisensory systems, the robot can deliver engaging experiences through visual, auditory, and motion-based feedback. This approach accommodates various sensory preferences and abilities, ensuring a more inclusive and accessible interaction for all elderly users.

## 7. Future Work

We have outlined several areas for improvement and suggests a roadmap for the next steps in this proposed work, including potential pilot studies and collaborations with healthcare institutions:

1. Miniaturization of the Integrated Structure. The goal is to miniaturize these components to fit within the frame of the humanoid robot, making the device more portable and providing a more personal and meaningful interaction for the elderly.

2. Expansion of Emotion Classification. Expanding the range of emotional expressions the robot can recognize to better meet the emotional needs of the elderly.

3. Pilot Studies and Collaboration. The paper implies future steps might involve conducting pilot studies and collaborating with healthcare institutions to test and refine the robot in real-world settings. These steps are critical for validating the effectiveness of the robot in providing emotional support to the elderly and ensuring it can be seamlessly integrated into healthcare environments.

4. Research and Development. Further research is necessary to enhance the robot’s capabilities, particularly in emotion recognition and interaction quality. This will likely involve iterative testing, user feedback, and technological upgrades.

5. Pet robots, like Paro [30,31] and Aibo [32,33], have been designed to offer companionship and emotional support, and they incorporate emotion detection technologies to interact more effectively with users. A detailed exploration of these examples, including their design, functionality, and effectiveness, would be in the scope of our future research work.

6. Ethical and Privacy Protection. Our future work incorporates robust data encryption and privacy measures to protect user data. In addition, it ensures compliance with data protection regulations and promotes user trust, essential for long-term sustainability in technology adoption [34,35]. Also to ensure that ethics are upheld accordingly to the IEEE standard [36,37].

By following this roadmap, this proposed work aims to create a more effective and empathetic companion robot that can significantly improve the emotional well-being of elderly users. Furthermore, to enhance the reliability and robustness of the data, our future work will focus on increasing the sample size. This approach will help ensure that the models are trained on a more diverse and representative dataset, thereby improving their generalizability and performance in real-world applications. By involving a larger and more varied group of participants, the project aims to better capture the range of facial expressions and emotions, leading to more accurate and effective facial expression analysis for the robot’s interactions with elderly users. To address potential ethical issues, this proposed work plans to implement two strategies. Firstly, its privacy protection. Ensuring that any data collected by the robot, such as facial expressions and other personal information, is handled with strict confidentiality. This involves employing robust data encryption methods and ensuring that the data is only accessible to authorized personnel. Regular audits and compliance with relevant data protection regulations will be conducted to maintain high privacy standards. Secondly, its mitigating emotional dependency by recognizing the risk of emotional dependency on robots, this proposed work aims to design interactions that promote healthy engagement without fostering over-reliance. The robot will be programmed to encourage social interactions with human caregivers and family members, thus acting as a complement rather than a replacement for human contact. Additionally, the content and responses generated by the robot will be carefully monitored to ensure they are appropriate and supportive rather than overly comforting or emotionally engaging. These measures are intended to balance the benefits of robotic assistance with ethical considerations, ensuring that the use of robots enhances the well-being of elderly users without compromising their privacy or emotional health.

At the same time, with life expectancy increasing, the demands on caregivers for the elderly are escalating while the availability of professionals in this field is dwindling and projected to continue declining. Consequently, this growing gap poses significant concerns for the foreseeable future. Fortunately, advancements in robotics and AI [30,34,35] have been progressing rapidly, enabling the execution of simple tasks to aid the elderly, such as constant heart rate monitoring with wearables and the use of autonomous wheelchairs for patient transport within facilities. While this proposed work does not directly address the physical needs of the elderly, it aims to tackle the hidden and often overlooked emotional challenges they face, particularly during periods of loneliness. By continuously monitoring the emotional well-being of the elderly through facial expression analysis and responding with compassionate behavior from a baby-sized robot, we aim not only to alleviate depression but also to uplift their spirits. As the proverb says, “a merry heart doeth good like a medicine” (Proverbs 17:22). There is still ample room for improvement in this work. Firstly, the Integrated Structure is currently too large for practical use, necessitating a miniaturization process. Ideally, the entire Integrated Structure, housing the Nvidia Jetson Nano platform, camera, interface controller, and power supply, should be condensed to fit within the humanoid robot’s frame. This approach would make the device highly portable and create a more personal and meaningful experience for the elderly, as they would interact solely with the humanoid robot. Secondly, instead of limiting the classification to just four types of expressions, expanding the range of classifications to include other emotional expressions could open up new possibilities for meeting additional emotional needs of the elderly. However, further research is needed to delve deeper into this aspect.

## Figures and Tables

**Table 1:** Summary of observations performed.

Subject	Expression Observed	Action from Robot	Feedback from Subject
A	Sad	Selects a dance routine	Feedback: Select a new story
B	Angry	Selects a story and executes	Feedback: Stopped robot

## Data Availability

The data presented in this study are available in this article.
